# Is there a role for citizen science in death and dying research?

**DOI:** 10.3389/fpubh.2023.1241239

**Published:** 2023-09-19

**Authors:** Clare Wilkinson, Alison Llewellyn, Candy McCabe

**Affiliations:** ^1^Science Communication Unit, College of Health, Science and Society, University of the West of England, Bristol, United Kingdom; ^2^Centre for Health and Clinical Research, College of Health, Science and Society, University of the West of England, Bristol, United Kingdom; ^3^Dorothy House Hospice, Winsley, United Kingdom

**Keywords:** hospice, death, dying, end-of-life care, public engagement, citizen science

## Abstract

The COVID-19 pandemic has brought conversations about death and dying to the fore in a way not experienced for generations. This raises questions around perceptions of death and dying; the role of healthcare and the community in care; and the use of digital media for information and support. Public engagement can provoke a two-way conversation between researchers and the public and includes techniques that can engage the community not only with the topic but also in research. This perspective article considers the potential role of citizen science in death and dying research, including considerations around its potential benefits and constraints.

## 1. Introduction

Previous studies of death and dying, and the role of hospices and palliative care, have found understanding among the public is mixed, with women and older people more commonly aware of the role of hospices (1). Lack of understanding can result in hospice care and community support being difficult to navigate (2), with hospice care underutilized, leading to calls for increased education and engagement (3, 4). Early conversations about death and dying are helpful in providing care and preparing for loss (5) but can be challenging conversations to have (2, 6, 7). In previous research conducted in the UK >75% of participants had some awareness/understanding around the role of hospices, but only half of these had a conversation about planning for their end-of-life (8). End-of-life care is increasingly focusing beyond professional settings, to family and “compassionate communities,” in promoting the wellbeing of dying, caring, and bereaved people (2, 9) therefore there may be increasing opportunities to engage and involve citizens in such conversations.

### 1.1. The role of public engagement

Death and dying are challenging and emotive subject areas, where there can be significant differences in understanding, alongside social, cultural, religious and spiritual variations. Public engagement has multiple definitions but is broadly understood to involve a two-way process, involving researchers and the public, which aspires to there being mutual benefit from the interaction (10).

Considerable research on death and dying has been conducted through traditional methods such as interviews and surveys (3), but these rarely include ‘public deliberation, whereby people engage collectively with an issue, consider it from all sides, and struggle to understand it’ (11). Challenges in public engagement around death and dying include resistance and lack of interest in planning ahead, a view that one is living rather than dying (even when experiencing significant or complex illness) and understanding how to demonstrate the relevance of the subject matter, beyond those experiencing death, dying or grief (12). Communicating relevance has become even more challenging when ‘public engagement on end-of-life cannot compete with the vast array of more powerful messages relating to youth and health’ (12).

Nevertheless, public engagement is increasing (13–17), including Death Cafes and festivals (18, 19) and via traditional (20), and social media (21). These engagement examples can be highly participatory in nature and build significantly on the lived experience. However, they also highlight the sensitivity of the subject, whereby differing opinions are common, and aspects of death and dying can be more challenging to discuss openly.

### 1.2. Citizen science

Citizen science is one of a vast array of public engagement methods now available to researchers and is broadly seen to be both an opportunity for participants to contribute to the scientific process and to gain something in return (22). Citizen science projects actively involve citizens in research that generates new knowledge or understanding, as contributors, collaborators or as project leaders (23). While it has an extensive history in fields such as natural history, archeology, and astronomy, the tools of social and digital media have particularly increased citizen sciences expansion, though many of the methods it uses are akin to epistemic approaches used by social scientists for many years (24).

Citizen science has been described as a “cluster of activities” which can include approaches such as open science and open innovation, drawing in a multitude of information sources and disciplines [(25), p. 1], but it also has important ramifications for stakeholder and community engagement in ‘building capacity for communities to participate in science and shape policy decision-making and implementation in the longer term’ [(25), p. 1].

People participate in citizen science for multiple reasons. These include personal and altruistic motivations, having interest and support for project goals or a desire to contribute to research, learn and participate (26). Notably, a number of the most successful citizen science projects, such as Galaxy Zoo (27) have tended to attract more male participants, and there are arguments that citizen science could be more inclusive (28). From the perspective of research however, citizen science can provide opportunities for people to collect and add data, participate in data processing and analysis, shape research questions or contribute to writing up. In ideal circumstances, citizen science projects create mutual benefit to both researchers and citizens, as well to the research itself (29).

Citizen science requires considerable advanced planning. This includes considering how participatory an activity will be, how it can be conducted safely and ethically, how data quality can be ensured and how participants will be adequately recognized within the process (26, 28). As a result of its increasing use a number of frameworks and principles for citizen science now exist. In Europe, the Ten Principles of Citizen Science set out a shared view of the characteristics that underpin high-quality citizen science (23). While in the US the Code on Crowdsourcing and Citizen Science Act (30) outlines citizen science and how it can be used in federal settings.

Moves to develop increased protocols and quality measures for citizen science may help to assist the approach to develop beyond the most obvious opportunities for data gathering and manipulation (31). In light of the growth of citizen science there are also calls for improved definitions of citizen science, including what it is, what it is not, and what quality criteria projects should meet (32).

## 2. Citizen science in health and social science research

The emergence of citizen science has arguably favored a science framing, neglecting the significant potential it may have in other areas of research including patient and public involvement in health settings, and the potential of the technique for use within the social sciences.

**Figure 1:**
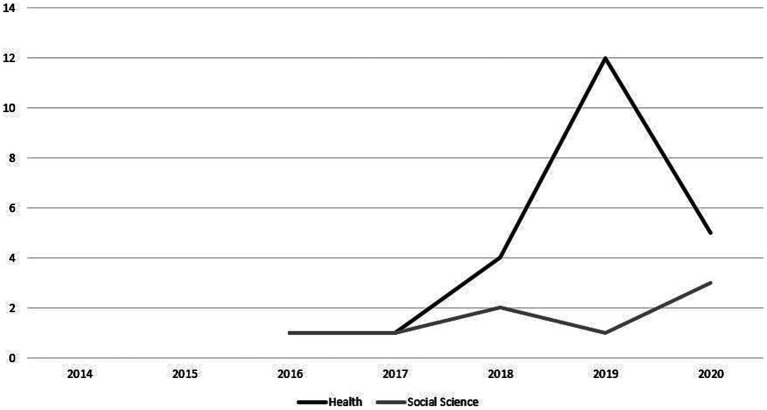
Articles by publication year.

To ascertain what is known about citizen science projects in these fields, we undertook a scoping review. Scoping reviews are helpful in determining whether a full systematic review is possible and aim to be transparent and systematic, but they do not operate with the same level of scope or assessment of quality (33). Two searches were conducted of the academic and grey literature in February 2020, including the terms “Citizen Science AND Health”, “Citizen Science AND Social Science” with search results then filtered and assessed for relevance. In the case of citizen science and health, references to environmental health were excluded. Editorials, commentaries excluding original data or projects, and letters in response to articles, were also excluded in both searches. The searches identified 23 relevant items located in the health search, and 10 in the social sciences search, including one item that appeared in both searches (Figure 1). The wider study in which this scoping review was conducted did not focus on assisted dying as a subject area. This topic was not excluded from the scoping review search criteria.

### 2.1. Citizen science in health research

The 23 items located within the context of health had all been published since 2016, with the majority published since 2018 (See Supplementary Table S1). Articles focused on use of health technologies and their potential adaptation for citizen science type projects (34). Public health was a popular area for articles, including focuses on emergency preparedness (35), the built environment (36), urban and rural environments (37), as well as broader questions around building public health and medical research that is more inclusive (38, 39). There were synthesis and scoping studies on issues including data management (40), as well as articles which attempted to create frameworks or models of different types of citizen science (41).

Articles stressed the relevance of citizen science for gathering health related data, for example using smart phones to capture photos and audio narratives (36), building in short surveys (42), interviewing (43) and the creation of dedicated apps (44). Benefits for citizens involved included personal empowerment as well as knowledge, skills and social networks (43). Benefits also extended to the communication of end results as the public have already been involved (42) and demonstrated the role citizen science could play in direct policy interventions (44). This also translated to ongoing actions and behavior changes, for example a citizen science project which included the recording of physical activity, resulted in the formation of a regular walking group (45).

It was common to explore the ethical challenges surrounding both citizen science and the capturing of health information (25), including how citizen scientists should be supported in sharing data and publishing findings, and the types of norms and policies that are emerging and can be adapted to health contexts (46).

### 2.2. Citizen science in social science research

Ten articles of relevance (See Supplementary Table S2) had been published since 2014, with the majority of articles published since 2018. The articles focused on topics including methods for citizen science, ethics, benefits and constraints, and use in specific disciplines such as computational science and workplace learning.

Articles discussed how citizen science can be amenable to the observation of everyday life, and therefore particularly responsive to the interests of social science and humanities researchers (47, 48). For example, observation of people requesting money on the street (49) or “pop up experiments” in urban settings (50), including approaches which are interdisciplinary (51). One article discussed the benefits for behavioral studies where lab-based experimental protocols have limitations (52). A further study endorsed the use of citizen science in workplace learning, in this case working with clinicians to gather data (53). Examples also discussed the relationship between citizen science as one form of participatory approach and behavior change, for example around environmental behaviors and climate change (54).

A number of articles discussed the benefits of citizen science for participants’ development, understanding and behavior, as well as social justice and scientific outcomes (55). Practical benefits included the opportunity to conduct research ‘*in situ*’ and to incorporate data collection and evaluation within the same tools (53). Combining citizen science approaches with big data was also seen to offer opportunities (56).

The relatively small number of articles, as well as content of a number of articles, suggested that citizen science has yet to be fully explored in social science contexts (26), or could be hidden within interdisciplinary projects where the scientific focus took most attention (47). Nevertheless, a number of benefits and constraints for citizen social science, as some articles described it, were identified.

## 3. Discussion

**Figure 2:**
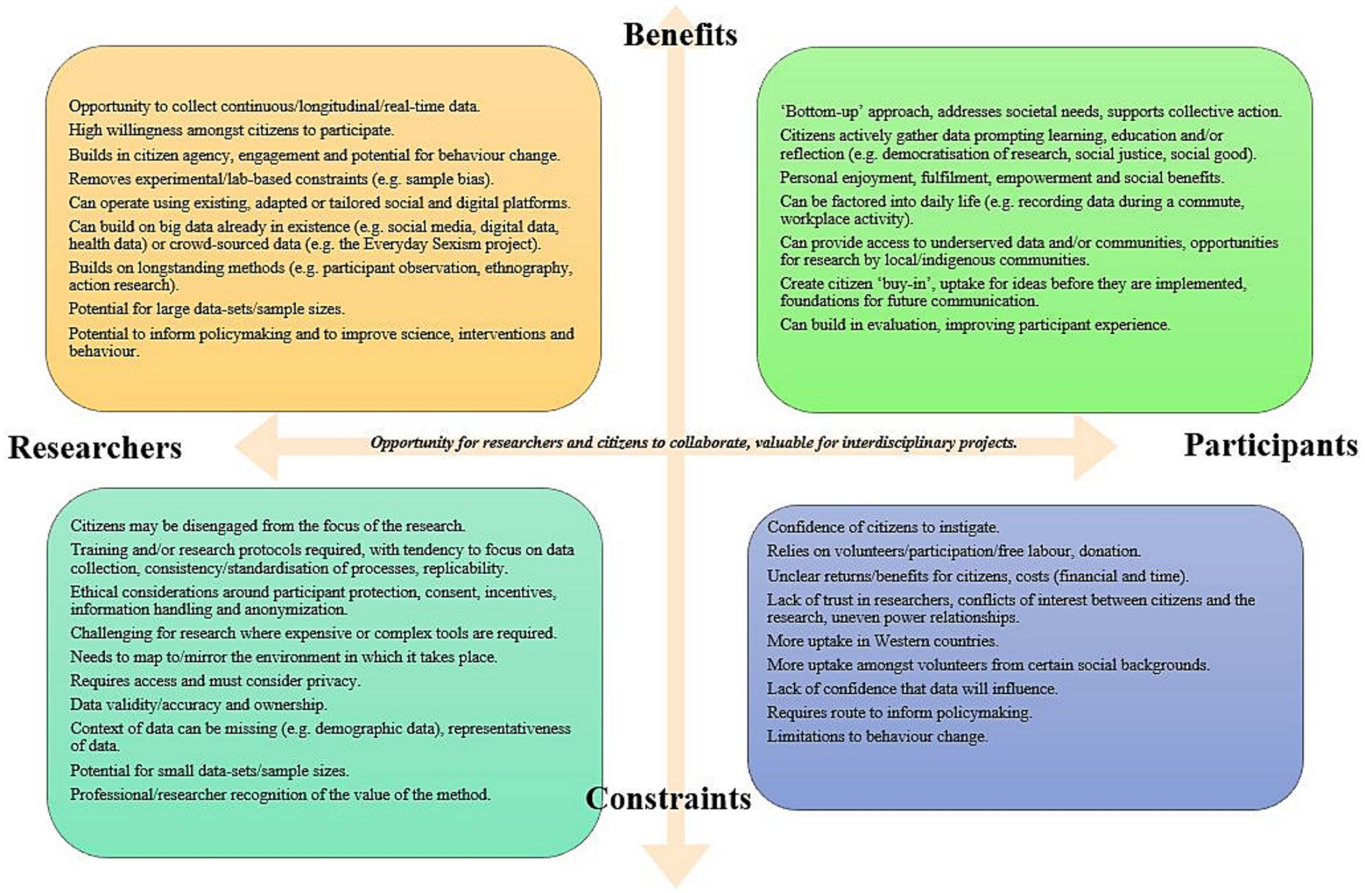
Benefits and constraints of citizen science for researchers and participants.

In summary, we found that there are emerging examples of projects taking place in both health and social science settings, which are utilizing a citizen science approach. Throughout the articles several key benefits and constraints in relation to citizen science were noted (Figure 2). These examples, and the literature resulting from them, suggests there are numerous opportunities to embed citizen science within a health or social science-based activity. These include citizen sciences potential to address societal needs, its benefits for participants and researchers, and applicability to situations that impact on our daily lives, making citizen science amenable to the context of research on death and dying.

For researchers, working on sensitive subject areas like death, dying, and end of life care, citizen sciences removal of lab-based and experimental settings provides opportunities to generate continuous data in real-life contexts, which could also include educative, therapeutic or supportive aspects for those participating (4). This may also mean such an approach can reach groups of people, who are otherwise underserved, for instance by working with communities when there are spiritual, social, or cultural variations in attitudes and practices toward death and dying, or encouraging men, for example, to engage in conversations around death and dying in gendered contexts (57).

Utilizing citizen science, including via social or digital media or community-based approaches, allows participants to contribute at times and in ways that are suited to their personal contexts, providing a level of empowerment and ownership, and also allowing citizens to shape and develop the approaches used. This may mean, in the right contexts, citizen science could work effectively around some of the most sensitive aspects of death and dying, for example the loss of infants and young children, or people who have died from suicide.

However, some of the benefits of citizen science in broader settings may not apply in the context of death and dying research. For example, digital and social media approaches may provide a sense of anonymity, which would be welcome for some participants and subject areas but feel impersonal or insensitive to others. This constraint may be particularly difficult given death and dying is a subject area where people already have a reluctance to share and converse (12). Similarly, while, citizen science works well in ‘everyday’ contexts and therefore offers benefits for integrating research on death and dying in social, community and family-based conversations, for those currently experiencing illness, the care of others, bereavement, or loss, it can already be a challenging and stressful time to seek to accommodate any kind of extra task.

There are clear constraints in citizen science, including considering ethical implications, the limitations of data, and appropriately recognizing the role of citizens in the research, which may be even more important around sensitive topics. Citizen science may not be well suited to such topics if the potential gain for those participating is unclear. Building trust would be essential and that would take time, and it would also be vital to ensure adequate support mechanisms were in place for the lived experiences and emotions that engagement on such topics can raise. From the researcher’s perspective this would require financial and time investment, to fully consider the ethical ramifications of a citizen science approach in this area and to make sure it was fully clear how any data was being used. One constraint which is arguably less applicable in the case of citizen science on death and dying is relevance, as the topic is relevant to all, but given the challenges in opening conversations in this area and indicating that relevance (12), creating uptake for such an activity, unless citizen led, would also require careful planning.

While our scoping review found that some projects and case studies included involved health conditions that could result in death and dying, it was evident there were no citizen science projects specifically focused on these topics, or end-of-life care at the time the review was undertaken. However, scoping reviews are limited in scope (33) and therefore further research would usefully ascertain the potential of a citizen science approach, as well as other public engagement techniques, in research on death and dying. To return to our original question, is there a role for citizen science in death and dying research, there is certainly potential, however any such approaches must proceed with time, care and compassion.

## Data availability statement

The original contributions presented in the study are included in the article/Supplementary material, further inquiries can be directed to the corresponding author.

## Ethics statement

The studies involving humans were approved by UWE Bristol University Ethics Committee. The studies were conducted in accordance with the local legislation and institutional requirements. Written informed consent for participation was not required from the participants or the participants’ legal guardians/next of kin in accordance with the national legislation and institutional requirements.

## Author contributions

CW, AL, and CM contributed to conception and design of the research. CW conducted the initial scoping review and wrote the first draft of the manuscript. AL and CM contributed to the scoping review analysis. All authors contributed to manuscript revision, read, and approved the submitted version.

## Funding

This work was supported by the College of Health, Science and Society, UWE Bristol.

## Conflict of interest

The authors declare that the research was conducted in the absence of any commercial or financial relationships that could be construed as a potential conflict of interest.

## Publisher’s note

All claims expressed in this article are solely those of the authors and do not necessarily represent those of their affiliated organizations, or those of the publisher, the editors and the reviewers. Any product that may be evaluated in this article, or claim that may be made by its manufacturer, is not guaranteed or endorsed by the publisher.
