# Supplementary material for: Is there a role for citizen science in death and dying research?
Source: Front Public Health. 2023 Sep 19;11:1241239. doi: 10.3389/fpubh.2023.1241239 (PMC10546016; doi:10.3389/fpubh.2023.1241239)
Supplement: Supplementary file 1 [file Table_1.DOCX]

Supplementary Material

Is there a role for Citizen Science in Death and Dying Research?

Clare Wilkinson*, Alison Llewellyn, Candy McCabe

*** Correspondence:** Corresponding Author: Clare.Wilkinson@uwe.ac.uk

**Table 1: Citizen Science and Health**

| Publication | Keywords | Description and Key Features |
| --- | --- | --- |
| 1. Bietz, M., Patrick, K, & Bloss, C. (2019) ‘Data Donation as a Model for Citizen Science Health Research’, *Citizen Science: Theory and Practice*, 4 (1).  <http://doi.org/10.5334/cstp.178> | Citizen science, data donation, informed consent, data access, privacy, data governance. | - New computational and sensing innovations, coupled with increasingly affordable access to consumer health technologies, allow individuals to generate personal health information that they are then able to submit to a shared archive or repository.  - Paper presents data donation as a model for health-focused citizen science, with special attention to the ethical challenges and opportunities that this model presents.  - Findings from the Health Data Exploration (HDE) Project’s second annual Network Meeting, include identification of four challenges for the ethical conduct of health-focused data donation research: Participant protection, representativeness, incentives to participate, and governance. |
| 2. Borda, A., Gray, K., & Fu, Y. (2019) ‘Research data management in health and biomedical citizen science: practices and prospects’, *JAMIA open*, 3(1), 113–125. <https://doi.org/10.1093/jamiaopen/ooz052> | Citizen science, crowdsourcing, participatory health, self-quantification, research data management | - Public engagement in health and biomedical research is being influenced by the paradigm of citizen science.  - A scoping review was conducted on this topic to determine data management characteristics of health and bio medical citizen science research.  - Health and biomedical citizen science platforms and projects are diverse in terms of types of work with data and data management activities that in themselves may have scientific merit. However, consistent approaches in the use of research data management models or practices seem lacking, or at least are not prevalent in the review.  - There is potential for important data collection and analysis activities to be opaque or irreproducible in health and biomedical citizen science initiatives without the implementation of a research data management model that is transparent and accessible to team members and to external audiences. |
| 3. Chari, R., Petrun Sayers, E.L., Amiri, S. et al. (2019) ‘Enhancing community preparedness: an inventory and analysis of disaster citizen science activities’, *BMC Public Health* 19, 1356. <https://doi.org/10.1186/s12889-019-7689-x> | No keywords present | - Disaster citizen science, or the use of scientific principles and methods by “non-professional” scientists or volunteers, may be a promising way to enhance public health emergency preparedness (PHEP) and build community resilience.  - To address research gaps, this paper: (1) assesses the state of disaster citizen science by developing an inventory of disaster citizen science projects; (2) identifies different models of disaster citizen science; and (3) assesses their relevance for PHEP.  - A final set of 209 projects, covering the time period 1953–2017, were included in the inventory. Projects were classified across five citizen science models: distributed or volunteer sensing (n = 19; 9%); contributory (n = 98; 47%); distributed intelligence (n = 52; 25%); collaborative research (n = 32; 15%); and collegial research (n = 8; 4%).  - By increasing engagement in research, disaster citizen science may empower communities to take collective action, improve system response capabilities, and generate relevant data to mitigate adverse health impacts. |
| 4. Chrisinger, B.W., & King, A.C. (2018) ‘Stress experiences in neighborhood and social environments (SENSE): a pilot study to integrate the quantified self with citizen science to improve the built environment and health’, *Int J Health Geogr* 17, 17. <https://doi.org/10.1186/s12942-018-0140-1>  *Also identified in Social Science AND Citizen Science* | No keywords present | - Developed a methodology to integrate geospatial technology with biometric sensing within a previously developed, evidence-based “citizen science” protocol, called “Our Voice.” Participants used a smartphone/tablet-based application, called the Discovery Tool (DT), to collect photos and audio narratives about elements of the built environment that contributed to or detracted from their well-being.  - 14 adults participated and recorded 174 images and 124 audio files with the DT.  - Participants identified a variety of social and environmental features that contributed to or detracted from their well-being. |
| 5. Katrien De Cocker, Sebastien F.M. Chastin, Ilse De Bourdeaudhuij, Ineke Imbo, Jeroen Stragier & Greet Cardon (2019) ‘Citizen Science to Communicate about Public Health Messages: The Reach of a Playful Online Survey on Sitting Time and Physical Activity’, *Health Communication*, 34:7, 720-725, <https://doi-org.ezproxy.uwe.ac.uk/10.1080/10410236.2018.1433955> | No keywords present | - There is a lack of research on how to communicate public health guidelines. Citizen science (CS) has been an effective way to involve the public in research.  - This study analyses the reach of a well-established CS experiment, launched during an annual national science event, to understand if it could be used as communication strategy for public health issues.  - A short playful online survey contained tailored health-related messages associated to an “animal totem” profile, based on the combination of sitting and physical activity levels (koala: high sitting, low activity; gorilla: high sitting, high activity; zebra: low sitting, low activity; bee: low sitting, high activity).  - A total of 6,246 adults completed the experiment, with a peak of views (n = 5,103) and completions (n = 1,209) a couple of days before the event.  - CS as part of a national science event is a good platform for health communication as 1 in 1,000 Flemish adults were reached. However, those completing the experiment were not representative of the general Flemish adult population and reported to be more physically active. |
| 6. Den Broeder, L., Kauw, K., Uysal, S., Schönenberger, M., Kwakkelstein-Klooster, S., Schoenmakers, M., Scharwächter, W., Schuit, A.J., & Wagemakers, A. (2016) ‘The Health Embassy. Resident benefits of Citizen Science in a low SES Dutch neighbourhood’, *European Journal of Public Health*, Volume 26, Issue suppl_1, , ckw171.016, <https://doi-org.ezproxy.uwe.ac.uk/10.1093/eurpub/ckw171.01> | No keywords present | - Slotermeer is a disadvantaged neighbourhood in Amsterdam, the Netherlands. Local policy makers want to collect information about resident views as a basis for health enhancing policies.  - A Citizen Science approach where residents interview people in the neighbourhood was applied to collect such information.  - Preliminary results show that the Health Ambassadors rated neighbourhood health more positively after the project. Self- reported knowledge and skills, in particular regarding health, were increased and personal social networks extended. Health Ambassadors developed a sense of urgency to continue working on improving neighbourhood health and have also put this to practice in various activities.  - Participating in Citizen Science yields personal empowerment for residents of a low-SES neighbourhood. It stimulates them to further engage in health enhancing activities. |
| 7. Den Broeder, L., Lemmens, L., Uysal, S., Kauw, K., Weekenborg, J., Schönenberger, M., Klooster-Kwakkelstein, S., Schoenmakers, M., Scharwächter, W., van de Weerd, A., El Baouchi, S., Schuit, A.J. & Wagemakers, A., (2017) ‘Public Health Citizen Science; Perceived Impacts on Citizen Scientists: A Case Study in a Low-Income Neighbourhood in the Netherlands’, *Citizen Science: Theory and Practice*, 2(1), p.7. <https://doi.org/10.5334/cstp.89> | Public health, community, disadvantaged groups, residents, citizen science experiences, neighbourhood | - Citizen science may yield crucial local knowledge and increase research capacity and there is growing interest to understand benefits for citizen scientists themselves.  - Studied the perceived impacts of participation in a public health citizen science project on citizen scientists in a disadvantaged neighbourhood in the Netherlands.  - Local citizen scientists, characterised by low income and low educational level – many of whom were of migrant origin – were trained to interview fellow residents about health-enhancing and health-damaging neighbourhood features.  - The results show that the citizen scientists perceived participation in the project as a positive experience. They acquired a broader understanding of health and its determinants and knowledge about healthy lifestyles, and took action to change their own health behaviour. They reported improved self-confidence and social skills, and expanded their network across cultural boundaries. Health was perceived as a topic that helped people with different backgrounds to relate to one another.  - Concludes that citizen science benefits participants with low educational or literacy level. Moreover, it seems to be a promising approach that can help promote health in underprivileged communities by strengthening personal skills and social capital. Embedding projects in broader health promotion strategies and long-term engagement of citizen scientists should be pursued to accomplish this. |
| 8. Den Broeder, L., Devilee, J., Van Oers, H., Schuit, A., J., & Wagemakers, A., (2018) ‘Citizen Science for public health’, *Health Promotion International*, 33 (3), 505–514 <https://doi-org.ezproxy.uwe.ac.uk/10.1093/heapro/daw086> | Public health, community participation, Citizen Science | - Public health policy is produced in a complex process resulting in policies that may appear not to link up to citizen perspectives.  - Addresses the central question as to whether citizen engagement in knowledge production could enable inclusive health policy making.  - Building on non-health work fields describes different types of citizen engagement in scientific research, or ‘Citizen Science’.  - Describes the challenges that Citizen Science poses for public health, and how these could be addressed, it may yield better knowledge, empowered communities, and improved community health.  - Provides a draft framework to enable evaluation of Citizen Science in practice, consisting of a descriptive typology of different kinds of Citizen Science and a causal framework that shows how Citizen Science in public health might benefit both the knowledge produced as well as the ‘Citizen Scientists’ as active participants. |
| 9. Fiske A, Prainsack B, & Buyx A (2019) ‘Meeting the needs of underserved populations: setting the agenda for more inclusive citizen science of medicine’, *Journal of Medical Ethics*, 45:617-622. <http://dx.doi.org.ezproxy.uwe.ac.uk/10.1136/medethics-2018-105253> | No keywords present | - In its expansion to genomic, epidemiological and biomedical research, citizen science has been promoted as contributing to the democratisation of medical research and healthcare.  - At the same time, it has been criticised for reinforcing patterns of exclusion in health and biomedicine, and sometimes even creating new ones.  - Although citizen science has the potential to make biomedical research more inclusive, the benefits of current citizen science initiatives are not equally accessible for all people—in particular those who are resource-poor, located outside of traditional networks of healthcare services, or members of minorities and marginalised groups.  - In view of growing public investments in participatory research endeavours, we argue that it should be considered more explicitly if, and how, citizen science could help make research more inclusive, contribute to the public good, and possibly even lead to better and more equitable healthcare.  - Proposes a set of relevant considerations for researchers, practitioners, bioethicists, funders and participants who seek to advance ethical practices of citizen-led health initiatives, and address profound differences in position, privilege and power in research. |
| 10. Frei, A., Dalla Lana, K., Radtke, T. et al. (2019) ‘A novel approach to increase physical activity in older adults in the community using citizen science: a mixed-methods study’, *Int J Public Health* 64, 669–678 <https://doi-org.ezproxy.uwe.ac.uk/10.1007/s00038-019-01230-3> | Physical activity, Walking intervention, Elderly Community, Citizen science, Long-term sustainability,  CAPACITY | - The aims of this study were to implement a novel, community-based physical activity (PA) intervention in a Swiss town with active participation of elderly participants and to evaluate its effectiveness, feasibility, acceptability and sustainability.  - The CAPACITY intervention combined important determinants of PA, used smartphone apps to provide feedback/facilitate interaction, and followed a citizen science approach to enable participants to organize walking groups.  - Twenty-nine persons were included in the study; 25 conducted 6-month follow-up. They had a significant increase in moderate-to-vigorous PA (p = 0.046) but not in daily steps (p = 0.331). After the intervention period, key participants took over organization, independently organized monthly get-togethers, added new walking routes and continuously recruit new participants. Eleven months after withdrawal of the study team, 61 people regularly walk in groups together. |
| 11. Gristwood A. (2019) ‘Public participation in science: How citizen science initiatives in healthcare and the environment are opening up new directions in research’, *EMBO reports*, 20(8), e48797. <https://doi.org/10.15252/embr.201948797> | No keywords present | - Discusses role of citizen science in environmental monitoring but that awareness of citizen science is not uniformly distributed across disciplines.  - Highlights need for increased understanding of citizen scientists, but that the approach can be used to reach groups with local knowledges.  - Provides example of DIY Biology project, Open Insulin, making insulin at home.  - Signposts to a potential role in patient engagement, including potential in pain management. |
| 12. Katapally, T. R., Bhawra, J., Leatherdale, S. T., Ferguson, L., Longo, J., Rainham, D., Larouche, R., & Osgood, N. (2018) ‘The SMART Study, a Mobile Health and Citizen Science Methodological Platform for Active Living Surveillance, Integrated Knowledge Translation, and Policy Interventions: Longitudinal Study’, *JMIR public health and surveillance*, 4(1), e31. <https://doi.org/10.2196/publichealth.8953> | Exercise, sedentary lifestyle, smartphone, ecological momentary assessments, epidemiological monitoring, translational medical research, health policy | - The primary objective of the Saskatchewan, let’s move and map our activity (SMART) Study was to develop a mobile and citizen science methodological platform for active living surveillance, knowledge translation, and policy interventions.  - This longitudinal investigation was designed to engage participants (ie, citizen scientists) in Regina and Saskatoon, Saskatchewan, Canada, in four different seasons across 3 years.  - In spring 2017, pilot data collection was conducted, where 317 adult citizen scientists (≥18 years) were recruited in person and online. Citizen scientists used a custom-built smartphone app, Ethica (Ethica Data Services Inc), for 8 consecutive days to provide a complex series of objective and subjective data.  - Pictures and audio files (ie, community voices) showed that the barriers and facilitators of active living included intrinsic or extrinsic motivations, social contexts, and outdoor or indoor environment, with pets and favorable urban design featuring as the predominant facilitators, and work-related screen time proving to be the primary barrier.  - The results also show the successful implementation of a platform that engages participants to catalyze policy interventions. Although SMART Study is currently geared toward surveillance, using the same platform, active living interventions could be remotely implemented. |
| 13. Katapally TR, & Chu LM (2020) ‘Digital epidemiological and citizen science methodology to capture prospective physical activity in free-living conditions: a SMART Platform study’, *BMJ Open* 2020;10:e036787. <http://dx.doi.org/10.1136/bmjopen-2020-036787> | No keywords present | - The purpose of this study was to develop a replicable methodology of mobile ecological momentary assessments (EMAs) to capture prospective physical activity (PA) within free-living social and physical contexts by leveraging citizen-owned smartphones running on both Android and iOS systems.  - 538 citizen scientists (≥18 years) provided PA data during eight consecutive days using a custom-built smartphone application, and after applying a rigid inclusion criteria, 89 were included in the final analysis.  - This digital epidemiological and citizen science methodology adapted mobile EMAs to capture not only prospective PA, but also important physical and social contexts within which individuals accumulate PA. |
| 14. King, A.C., Winter, S.J, Chrisinger, B.W., Hua, J., & Banchoff, A.W. (2019) ‘Maximizing the promise of citizen science to advance health and prevent disease’, *Preventive Medicine*, 119, 44-47.  <https://doi.org/10.1016/j.ypmed.2018.12.016>. | Citizen science, Community engagement, Population health, Built environment | - “Citizen science” has gained increasing interest in medical and broader health fields both in the U.S. and internationally.  - Efforts to engage community members in the scientific process can demystify science and make it more accessible and inclusive and aid in advancing biomedical and other health-related discoveries and impacts.  - In light of the aging U.S. population, escalating health costs, and widespread health disparities, leveraging the power of the public as change agents in promoting health-enhancing conditions can complement the work of health professionals.  - Maximizing the advantages that citizen science offers the health sciences requires expansion of thought and action beyond the more limited definitions currently being emphasized in the U.S. health arena. |
| 15. Lehman, E., Jepson, R., McAteer, J., & Archibald, D. (2020) ‘What Motivates Volunteers to Engage in Health-Related Citizen Science Initiatives? A Case Study of Our Outdoors’, *International journal of environmental research and public health*, 17(19), 6950. <https://doi.org/10.3390/ijerph17196950> | Citizen science, environmental volunteering, motivations, public health | - Citizen science is increasing in popularity but remains largely located in the disciplines of environmental and natural sciences.  - The study aimed to identify the factors for involvement (or non-involvement) in health-related citizen science projects using the Our Outdoors citizen science initiative.  - Our Outdoors aims to understand how urban and rural shared outdoors spaces (e.g., parks, lakes, rivers, beaches) can affect human health and well-being (both positively and negatively).  - A thematic analysis revealed five key themes. These include enhancing social connectedness; personal learning development; making a difference in the community; gaining health and well-being benefits; and finally, demotivating factors relating to time constraints and the term “citizen science”.  - This study concludes that emphasising motivating factors in the promotional material for health-related citizen science projects may increase recruitment and the active involvement of participants. Similarly, reducing the presence of demotivating factors and considering the use of the term “citizen science” is likely to encourage participation. |
| 16. Mac Domhnaill, C., Lyons, S. & Nolan, A., (2020) ‘The Citizens in Citizen Science: Demographic, Socioeconomic, and Health Characteristics of Biodiversity Recorders in Ireland’, *Citizen Science: Theory and Practice*, 5(1), p.16. http://doi.org/10.5334/cstp.283 | Citizen science, biodiversity recorders, attitudes, demography, health, Ireland | - Design of recruitment campaigns and selection of activities for citizen science projects could benefit from a better understanding of participants’ demographic and socioeconomic characteristics, as well as participants’ health, their well-being status, and their level of physical activity.  - This type of information can provide a baseline for examining effects of engaging in citizen science on participants’ health and well-being in later research.  - Reports results from a survey of a group of environmental citizen scientists engaged in observation and monitoring activities who have recently registered as biodiversity data recorders in Ireland.  - Results show that biodiversity recorders are more highly educated, more middle-aged, more rural, better-off, and more active in the labour force than the general population.  - Biodiversity recorders are more physically active than the wider population, and mixed results are found for generalised health and mental health status. |
| 17. Majumder MA & McGuire AL. (2020) ‘Data Sharing in the Context of Health-Related Citizen Science’, *The Journal of Law, Medicine & Ethics*, 48(1_suppl), 167-177.  <https://doi-org.ezproxy.uwe.ac.uk/10.1177/1073110520917044> | No keywords present | - As citizen science expands, questions arise regarding the applicability of norms and policies created in the context of conventional science.  - This article focuses on data sharing in the conduct of health-related citizen science, asking whether citizen scientists have obligations to share data and publish findings on par with the obligations of professional scientists.  - Concludes that there are good reasons for supporting citizen scientists in sharing data and publishing findings. At the same time, it is problematic to treat data sharing and publication as ethical requirements for citizen scientists, especially where there is the potential for burden and harm without compensating benefit. |
| 18. Ottaviano M, Beltrán-Jaunsarás ME, Teriús-Padrón JG, García-Betances RI, González-Martínez S, Cea G, Vera C, Cabrera-Umpiérrez MF, Arredondo & Waldmeyer MT. (2019) Empowering Citizens through Perceptual Sensing of Urban Environmental and Health Data Following a Participative Citizen Science Approach. *Sensors,* 19(13):2940. <https://doi.org/10.3390/s19132940> | Citizen science; pollution; public health; environmental sensors; sustainable lifestyle; green behaviour; user empowerment | - This paper presents the design and development of a novel participatory citizen science-based application and data ecosystem model.  - This data acquisition approach allows citizens to gather and generate environment- and health-related data through mobile devices. The sum of all citizens’ data will continuously enrich and increase the volumes of data coming from the city sensors and sources across geographical locations.  - Although it is difficult for citizens to relate their personal behaviour to large-scale problems such as climate change, pollution, or public health, the developed ecosystem provides the necessary tools to enable a greener and healthier lifestyle, improve quality of life, and contribute towards a more sustainable local environment. |
| 19. Pomeroy, S. J., Minaker, L. M., & Mah, C. L. (2018). ‘An exploration of citizen science for population health research in retail food environments’, *Canadian journal of public health*, 108(5-6), e636–e638. <https://doi.org/10.17269/cjph.108.6099> | Public health practice, environment and public health, community participation | - Describes how citizen science approaches were incorporated into a public engagement activity as part of a population health intervention research project on the retail food environment, a workshop called The Food In This Place in St. John’s, Newfoundland and Labrador.  - Used citizen science methods and approaches to train and support participants to critically analyze a sample of everyday local retail food environments. |
| 20. Rappold, A.G., Hano, M.C., Prince, S., Wei, L., Huang, S.M., Baghdikian, C., Stearns, B., Gao, X., Hoshiko, S., Cascio, W.E., Diaz‐Sanchez, D., & Hubbell, B. (2019) ‘Smoke Sense Initiative Leverages Citizen Science to Address the Growing Wildfire‐Related Public Health Problem’ *GeoHealth*, 3(12) 443-457. <https://doi.org/10.1029/2019GH000199> | Citizen Science, Health Behavior, Wildfire Smoke, Smartphone App | - Smoke Sense is a citizen science project with investigative, educational, and action‐oriented objectives at the intersection of wildland fire smoke and public health.  - Participants engage with a smartphone application to explore current and forecast visualizations of air quality, learn about how to protect health from wildfire smoke, and record their smoke experiences, health symptoms, and behaviors taken to reduce their exposures to smoke.  - Data from the pilot season (1 August 2017 to 7 January 2018; 5,598 downloads) suggest that there is a clear demand for personally relevant data during wildfire episodes motivated by recognition of environmental hazard and the personal concern for health. However, while participants shared clear perceptions of the environmental hazard and health risks in general, they did not consistently recognize their own personal health risk. The engagement in health protective behavior was driven in response to symptoms rather than as preventive courses of action. |
| 21. Walls, T. A., Forkus, S. & Coria, A. (2019) ‘Citizen Health Science’, *International Journal of Population Data Science*, 4 (1).  <https://doi.org/10.23889/ijpds.v4i1.1074> | Citizen health science, single-case designs, single-subject studies, idiographic research, self-experimentation. | - Citizen scientists with health interests have become more equipped to conduct their own types of N-of-1 studies given new tools such as smart phone tracking and many other mobile health or “mHealth” devices.  - These advances in the single case or N-of-1 tradition sometimes involve self-tracking of health data and/or self-experimentation with one’s own health.  - As networks (increasingly, virtual ones) of people work to maintain or improve their health, a new platform for scientific inquiry has emerged. |
| 22. Wiggins, A. & Wilbanks, J. (2019) ‘The Rise of Citizen Science in Health and Biomedical Research’, *The American Journal of Bioethics*, 19:8, 3-14.  <https://doi.org/10.1080/15265161.2019.1619859> | Citizen science, confidentiality and privacy, participatory research, professional–patient relationship, research ethics, social science research. | - Citizen science models of public participation in scientific research represent a growing area of opportunity for health and biomedical research, as well as new impetus for more collaborative forms of engagement in large-scale research.  - Citizen science raises a variety of ethical issues that both fall outside of and build upon the standard human subjects concerns in bioethics.  - Provides background on citizen science, examples of current projects in the field, and discussion of established and emerging ethical issues for citizen science in health and biomedical research. |
| 23. van den Berge, M., Hulsegge, G., van der Molen, H. F., Proper, K. I., Pasman, H., den Broeder, L., Tamminga, S. J., Hulshof, C., & van der Beek, A. J. (2020) ‘Adapting Citizen Science to Improve Health in an Occupational Setting: Preliminary Results of a Qualitative Study’, *International journal of environmental research and public health*, 17(14), 4917. <https://doi.org/10.3390/ijerph17144917> | Blue-collar workers, worksite health promotion, unhealthy lifestyle, citizen science | - Citizen science engages target groups in the design and execution of health interventions, but has not yet been applied in an occupational setting.  - This preliminary study determines barriers and facilitators and feasible elements for citizen science to improve the health of blue-collar workers.  - Workers considered work pressure, work location and several personal factors as barriers for citizen science at the worksite, and (lack of) social support and (negative) social culture both as barriers and facilitators.  - Citizen science to improve health at the worksite may include three elements: (1) knowledge and skills, (2) social support and social culture, and (3) awareness about lifestyle behaviors. Strategies to implement these elements may be company specific. |

**Table 2: Citizen Science and Social Science**

| Publication | Keywords | Description and Key Features |
| --- | --- | --- |
| 1. Purdam K. (2014) Citizen social science and citizen data? Methodological and ethical challenges for social research. *Current Sociology*, 62(3), 374-392. <https://doi.org/10.1177/0011392114527997> | Citizen, crowd-source, emancipatory social science, observation, crowd-sourcing | - Many citizens are already creating digital data archives of their own lives through online activity including via social media communication. Citizens now have the potential to be the default fieldworkers of their own lives. This can be extended to examine the value of citizens systematically collecting data on the world around them for social science research.  - Pilot observation study required volunteers to follow a protocol and record the number of people seen begging.  - The study produced important findings on begging which informed a larger research project. However, challenging methodological and ethical issues are raised concerning the observation of public life. Even so, it is clear there is potential for what can be termed ‘citizen social science’. |
| 3. Vicens, J., Perelló, J., & Duc, J. (2018) Citizen Social Lab: A digital platform for human behavior experimentation within a citizen science framework. *PLoS ONE*, 13(12), e0207219. <https://doi.org/10.1371/journal.pone.0207219> | No keywords present | - Behavioral experiments have been largely conducted to shed light into the mechanisms behind cooperation—and other behavioral traits. However, most of these experiments have been conducted in laboratories with highly controlled experimental protocols but with limitations in terms of subject pool or decisions’ context, which limits the reproducibility and the generalization of the results obtained.  - Introduces Citizen Social Lab, a software platform designed to be used in the wild using citizen science practices. The platform allows researchers to collect data in a more realistic context while maintaining the scientific rigor, and it is structured in a modular and scalable way so it can also be easily adapted for online or brick-and-mortar experimental laboratories.  - Following citizen science guidelines, the platform is designed to motivate a more general population into participation, but also to promote engaging and learning of the scientific research process. |
| 4. Makuch, KE, & Aczel MR. (2020) Eco-Citizen Science for Social Good: Promoting Child Well-Being, Environmental Justice, and Inclusion. *Research on Social Work Practice*, 30(2), 219-232. <https://doi-org.ezproxy.uwe.ac.uk/10.1177/1049731519890404> | Environmental justice, environmental citizen science, children citizen science, sustainability, social good | - Article examines the benefits and challenges of engaging children in environmental citizen science, defined as science conducted by non-specialists under the direction of professional scientists, to promote social good.  - Article evaluates how participation in citizen science projects focused on the environment (eco-citizen science) benefits the child’s development, contributes to science, and leads to commitment to environmental stewardship and justice as adults. – The work offers a novel contribution to the discourse on social good and social justice through explicitly calling for children to be included in environmental citizen science projects.  - Examines the benefits and challenges of involving children in scientific projects and discuss implications for policy, practice, and future research. |
| 5. Dadich, A. (2014) Citizen social science: a methodology to facilitate and evaluate workplace learning in continuing interprofessional education. *Journal of Interprofessional Care*, 28:3, 194-199. <https://doi-org.ezproxy.uwe.ac.uk/10.3109/13561820.2013.874982> | Continuing interprofessional education, crowdsourcing, social media, workplace learning, youth health | - Workplace learning in continuing interprofessional education (CIPE) can be difficult to facilitate and evaluate, which can create a number of challenges for this type of learning. This article presents an innovative method to foster and investigate workplace learning in CIPE – citizen social science.  - Citizen social science involves clinicians as co-researchers in the systematic examination of social phenomena. When facilitated by an open-source online social networking platform, clinicians can participate via computer, smartphone, or tablet in ways that suit their needs and preferences. Furthermore, as co-researchers they can help to reveal the dynamic interplay that facilitates workplace learning in CIPE.  - Although yet to be tested, citizen social science offers four potential benefits: it recognises and accommodates the complexity of workplace learning in CIPE; it has the capacity to both foster and evaluate the phenomena; it can be used in situ, capturing and having direct relevance to the complexity of the workplace; and by advancing both theoretical and methodological debates on CIPE, it may reveal opportunities to improve and sustain workplace learning. |
| 6. Sagarra, O, Gutiérrez-Roig, M, Bonhoure, I., & Perelló, J, (2016) Citizen Science Practices for Computational Social Science Research: The Conceptualization of Pop-Up Experiments. Frontiers in Physics, 3:93. <https://doi.org/10.3389/fphy.2015.00093> | Citizen Science, participation, engagement, computational social science, data, experiments, collective, methods | - Citizen Science can furnish ready-made solutions with citizens playing an active role. However, this framework is still far from being well established as a standard tool for computational social science research.  - Presents experience in bridging gap between computational social science and the philosophy underlying Citizen Science, which in our case has taken the form of “pop-up experiments.” These are non-permanent, highly participatory collective experiments which blend features developed by big data methodologies and behavioral experimental protocols with the ideals of Citizen Science.  Explains the solutions implemented, providing practical examples grounded in experience in an urban context (Barcelona, Spain).  - This work will serve as a guideline for groups willing to adopt and expand such in vivo practices and opens up the debate regarding the possibilities (and also the limitations) that the Citizen Science framework can offer the study of social phenomena. |
| 7. Banerjee, S. (2018) Citizen Data Science For  Social Good In Complex Systems. *Interdisciplinary Description of Complex Systems,* 16(1), 88-91. <https://doi.org/10.7906/indecs.16.1.6> | Citizen data, Zenodo, complex systems | - The confluence of massive amounts of openly available data, sophisticated machine learning algorithms and an enlightened citizenry willing to engage in data science presents novel opportunities for crowd sourced data science for social good.  - Presents vignettes of data science projects which have impact in various spheres of life and on social good.  - Complex systems are all around us: from social networks to transportation systems, cities, economies and financial markets. Understanding these complex systems may lead to solutions for problems ranging from famines, global crises, poverty, climate change and sustainable living despite over-population.  - Big data and citizen data science allows unprecedented computational power and collective intelligence to be brought to bear on fundamental challenges facing humanity like poverty, diseases, famines and developmental challenges. |
| 8. Pykett, J., Chrisinger, B., Kyriakou, K. et al. (2020) Developing a Citizen Social Science approach to understand urban stress and promote wellbeing in urban communities. *Palgrave Communications* 6, 85. <https://doi.org/10.1057/s41599-020-0460-1> | No keywords present | - Paper sets out the future potential and challenges for developing an interdisciplinary, mixed-method Citizen Social Science approach to researching urban emotions. It focuses on urban stress, which is increasingly noted as a global mental health challenge facing both urbanised and rapidly urbanising societies.  - The paper reviews the existing use of mobile psychophysiological or biosensing within urban environments—as means of ‘capturing’ the urban geographies of emotions.  - In comparing perspectives on the conceptualisation and measurement of urban stress from psychology, neuroscience and urban planning, the difficulties of defining scientific constructs within Citizen Science are discussed to set out the groundwork for fostering interdisciplinary dialogue.  - The novel methods, geo-located sensor technologies and data-driven approaches to researching urban stress now available to researchers pose a number of ethical, political and conceptual challenges around defining and measuring emotions, stress, human behaviour and urban space. They also raise issues of rigour, participation and social scientific interpretation.  - Introducing methods informed by more critical Citizen Social Science perspectives can temper overly individualised forms of data collection to establish more effective ways of addressing urban stress and promoting wellbeing in urban communities. |
| 9. Tauginienė, L., Butkevičienė, E., Vohland, K. et al. (2020) Citizen science in the social sciences and humanities: the power of interdisciplinarity. *Palgrave Communication* 6, 89. <https://doi.org/10.1057/s41599-020-0471-y> | No keywords present | - Citizen science evolved through multiple disciplinary manifestations into a new field of study and a participatory method of enquiry.  - While most citizen science projects take place within problem-focused natural sciences, social sciences and humanities help understanding the human dimension and open a broad methodological spectrum for enriching scientific research with new approaches and for boosting public participation.  - Uses a meta-synthesis approach to explore how citizen science is practised in the so far less addressed social sciences and humanities by focusing on the role of the citizens, the goals and approaches of the projects, the tasks in which citizens are engaged and their gains across projects of diverse disciplinary background.  - Findings indicate that social sciences are gaining more acknowledgment within interdisciplinary citizen science projects by addressing ‘wicked’ problems of human behaviour and agency, while humanities are in quest of a better-defined locus in citizen science.  - Concludes that social sciences and humanities still face considerable barriers to infiltrate citizen science; the payoffs are substantial and already rewarding for several subfields in social sciences and humanities. |
| 10. Heiss, R. & Matthes, J. (2017) Citizen Science in the Social Sciences: A Call for More Evidence. *GAIA - Ecological Perspectives for Science and Society*. 26. 22-26. 10.14512/gaia.26.1.7. | Citizen science, co-production of knowledge, public participation in scientific research, social sciences | - While citizen science research is already established in the natural sciences, it remains difficult to find in mainstream social sciences. Yet, social science research stands to benefit greatly from civic engagement.  - In this age of new technologies, citizen contributions have become more feasible. In Austria, for example, students are engaged in a citizen science project called YAPES, in which they use their smartphones to submit comments and photos about how they get in touch with political issues.  - Challenges do exist for this form of research, however, from the relation between human observation and data quality to ethical questions. |
